# Effect of Hydroxychloroquine on the Retinal Layers: A Quantitative Evaluation with Spectral-Domain Optical Coherence Tomography

**DOI:** 10.1155/2016/8643174

**Published:** 2016-08-30

**Authors:** Hasim Uslu, Bulent Gurler, Aydin Yildirim, Mehmet Gurkan Tatar, Feride Aylin Kantarcı, Hasan Goker, Hatice Seval Pehlevan, Hatice Nur Colak

**Affiliations:** ^1^Department of Ophthalmology, Fatih University Medical Faculty Hospital, 34844 Istanbul, Turkey; ^2^Department of Rheumatology, Fatih University Medical Faculty Hospital, 34844 Istanbul, Turkey

## Abstract

*Purpose.* To evaluate the effect of hydroxychloroquine on retinal pigment epithelium- (RPE-) Bruch's membrane complex, photoreceptor outer segment, and macular ganglion cell-inner plexiform layer (GCIPL) thicknesses using spectral-domain optical coherence tomography (SD-OCT).* Methods.* In this prospective case-control study, 51 eyes of 51 hydroxychloroquine patients and 30 eyes of 30 healthy subjects were included. High-quality images were obtained using a Cirrus HD-OCT with 5-line raster mode; the photoreceptor inner segment (IS) and outer segment (OS), sum of the segments (IS + OS), and RPE-Bruch's membrane complex were analyzed.* Results.* The thicknesses of the IS + OS and OS layers were significantly lower in the hydroxychloroquine subjects compared to the control subjects (*P* < 0.05). RPE-Bruch's membrane complex thicknesses were significantly higher in the hydroxychloroquine subjects than for those of the control subjects (*P* < 0.05). The minimum and temporal-inferior macular GCIPL thicknesses were significantly different between the patients with hydroxychloroquine use and the control subjects (*P* = 0.04 and *P* = 0.03, resp.).* Conclusions.* The foveal photoreceptor OS thinning, loss of GCIPL, and RPE-Bruch's membrane thickening were detected in patients with hydroxychloroquine therapy. This quantitative approach using SD-OCT images may have important implications to use as an early indicator of retinal toxicity without any visible signs of hydroxychloroquine retinopathy.

## 1. Introduction

Hydroxychloroquine is a commonly used immunosuppressive agent in the treatment of various autoimmune diseases. Despite lesser systemic toxicity compared to other drugs, it can cause severe retinal dysfunction and loss of vision. Retinal toxicity can occur as a side effect of long-term hydroxychloroquine therapy. The most important risk factors are high dose and long duration of use. Dosage > 5.0 mg/kg dramatically increases both population risk and annual incremental risk, and extreme doses can be exceedingly dangerous. Other major factors are concomitant renal disease or use of tamoxifen [1, 2]. Those who advocate universal screening for hydroxychloroquine retinopathy do so because they believe that the best estimate for prevalence of hydroxychloroquine toxicity is greater than 1% among all users of these drugs for more than 5 years [3].

Although hydroxychloroquine retinopathy can be asymptomatic in early stages, patients with more advanced toxicity progress to having night vision problems and paracentral scotomas [4]. This change has been characterized typically as photoreceptor thinning that begins in a parafoveal ring and progresses over time to become a visible bull's-eye retinopathy when the retinal pigment epithelium (RPE) becomes damaged. The progression of damage of structural and functional deficits can occur even after the cessation of drug therapy [5]. Discontinuing the drug in the early stages can prevent permanent damage; therefore, the screening of patients for the early detection of asymptomatic retinal structural changes is important [6].

In 2016, the American Academy of Ophthalmology published recommendations on screening for chloroquine and hydroxychloroquine retinopathy. The primary screening tests are automated visual fields plus spectral-domain optical coherence tomography (SD-OCT). The multifocal electroretinogram (mfERG) can provide objective corroboration for visual fields, and fundus autofluorescence (FAF) can show damage topographically. Modern screening should detect retinopathy before it is visible in the fundus [2]. Of these tests, SD-OCT is the most commonly used device for the detection of retinal structural changes. Therefore, in our design of this study to investigate the early signs of retinal toxicity, we aimed to detect early abnormalities of the photoreceptor inner segment (IS), outer segment (OS), and RPE-Bruch's membrane complex thicknesses using SD-OCT image segmentation algorithms.

## 2. Methods

### 2.1. Study Design, Setting, and Population

This prospective case-control study was performed at the Ophthalmology Department of Fatih University Medical Faculty Hospital and conducted according to the principles of the Declaration of Helsinki. The study was approved by the Institutional Review Board of the University of Fatih, and informed consent was obtained from all participants.

A total of 81 participants were included in this study. Participants were divided into two groups: Group 1 (hydroxychloroquine use) and Group 2 (control). Participants in the hydroxychloroquine use group were chosen from patients being treated for rheumatic diseases, including rheumatoid arthritis, systemic lupus erythematosus, and Sjögren's disease. Control subjects were selected from healthy volunteers.

Additionally, Group 1 was divided into two subgroups based on the duration of hydroxychloroquine treatment as follows: 26 patients with hydroxychloroquine use <5 years and 25 patients with hydroxychloroquine use ≥5 years.

All subjects were excluded if they had histories of glaucoma or glaucoma suspects, uveitis, optic neuropathy, or retinal or choroidal vascular disease; refractive error of more than ±4 D sphere or ±2 D cylinder; or had undergone previous ocular surgery.

Clinical information included adjusted daily dose of drug, duration of drug exposure, and cumulative dose of drug. Adjusted daily dose was calculated as described by Browning [7] (daily dose divided by the lesser of the ideal body weight (IBW) associated with the patient's height or the actual body weight (ABW)). The hydroxychloroquine cumulative dose (g) was calculated by multiplying the current daily dose (g/day) with the duration of hydroxychloroquine treatment (month).

### 2.2. Ocular Examination and Psychophysical Tests

All participants underwent a comprehensive ocular examination, including visual acuity, slit-lamp biomicroscopy, intraocular pressure (IOP) measurement with Goldmann applanation tonometry, central corneal thickness (CCT) measurement with a Pentacam Scheimpflug (Oculus Inc., Wetzlar, Germany), axial length (AL) measurement with the IOLMaster (Carl Zeiss, Jena, Germany), and pupil-dilated fundus examination. In addition, subjects with hydroxychloroquine use underwent white-on-white visual field (VF) testing using the Humphrey macular threshold program (Zeiss Humphrey Systems, Dublin, CA).

### 2.3. SD-OCT Examination

The Cirrus HD-OCT 4000, version 6.0, ganglion cell analyzer (GCA) algorithm was used to detect the GCIPL and to measure the thickness of the elliptical annulus area (vertical outer radius 2.0 mm, horizontal outer radius 2.4 mm) centered on the fovea. The foveal region was not included in the measurement. The GCA reports the average, minimum, and sectoral (superotemporal, superior, superonasal, inferonasal, inferior, and inferotemporal) thicknesses of the GCIPL. The Cirrus HD 5-line raster mode was used to obtain high-quality images. The scan lengths were 6 mm for the horizontal scans and 6 mm for the vertical scans. The thicknesses were measured at the fovea and at 0.5 mm nasal, temporal, inferior, and superior to the fovea. The thicknesses were manually measured using the software loaded in the system by the examiner who was unaware of the hydroxychloroquine history of the subjects. The signal strength ranged from 1 to 10. The scans of strengths 1–7 were excluded from the study.

### 2.4. Mean Outcome Measures

**Figure 1:**
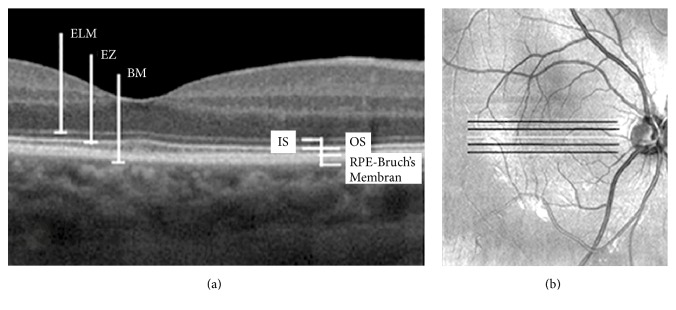
Spectral-domain optical coherence tomography (SD-OCT) horizontal scan (passing through the foveal center) image of a healthy right eye. High-definition imaging with SD-OCT allows detailed description of the retinal structures. The retinal layers are labeled from the top to the bottom as external limiting membrane (ELM), ellipsoid zone (EZ), and retinal pigment epithelium- (RPE-) Bruch's membrane complex.

The thicknesses of the photoreceptor IS and OS and IS + OS at the fovea were analyzed [8]. High-definition image segmentation was considered as follows (Figure 1).

 IS thickness is the distance between the lower border of the external limiting membrane (ELM) and the middle of the ellipsoid zone (EZ).
 OS thickness refers to the distance between the middle of the EZ and the anterior surface of the RPE.
 IS + OS thickness accounts for the distance between the lower border of the ELM and the anterior surface of the RPE.
 RPE-Bruch's membrane complex thickness refers to the distance between the lower border of Bruch's membrane and the anterior surface of the RPE.

### 2.5. Statistical Analysis

Statistical analysis was performed with SPSS software for Windows, version 18 (SPSS Inc., Chicago, IL, USA). Only the right eye of each participant was included for analysis. The distributions of continuous variables were determined by a Kolmogorov-Smirnov test. Homogeneity of variances was evaluated using a Levene test. Thickness measurements in the central macular areas obtained from images in Group 1 (hydroxychloroquine patients) were compared to those obtained in Group 2 (controls). The mean differences among groups were analyzed using a Student's* t*-test. The correlation between the exposure time, cumulative dose, and SD-OCT parameters was evaluated using Pearson's correlation coefficient. The data are expressed as the mean ± standard deviation for the continuous variables. *P* values < 0.05 were considered statistically significant. We performed a multiple-adjusted testing analysis (Bonferroni method) to evaluate the influence of the photoreceptor IS and OS, IS + OS, and RPE-Bruch's membrane complex thicknesses.

## 3. Results

### 3.1. Demographic Results

The demographic characteristics and clinical data of the patients receiving hydroxychloroquine and the control subjects are shown in Table 1. The mean age of the 51 patients was 45.0 ± 13.16 years in hydroxychloroquine patients and 42.1 ± 14.3 years in the controls (*P* = 0.35). The mean cumulative dose of hydroxychloroquine was 648.41 g, and the mean duration of hydroxychloroquine use was 34.85 months. The mean adjusted daily dosing was 8.75 mg/kg/d. There were no statistically significant differences in IOP, AL, CCT, and BMI between the groups (*P* = 0.18, *P* = 0.95, *P* = 0.25, and *P* = 0.58, resp.). Indications for hydroxychloroquine use included 20 cases for rheumatoid arthritis, 19 cases for systemic lupus erythematosus, and 12 cases for Sjögren's syndrome.

### 3.2. SD-OCT Findings

The OS and IS + OS thicknesses of hydroxychloroquine patients were lower than those of the control subjects and were significantly different at all five locations that were assessed (*P* < 0.003). The IS thicknesses were not significantly different between the patients with hydroxychloroquine use and the control subjects (*P* > 0.05). RPE-Bruch's membrane complex thicknesses for using hydroxychloroquine were also significantly higher than for those of the control subjects (Table 2).

The GCIPL thickness values of the hydroxychloroquine and control groups are compared in Table 3. Minimum and temporal-inferior macular GCIPL thicknesses were 79.73 ± 5.07 and 81.78 ± 5.44 *μ*m in hydroxychloroquine patients, and 81.97 ± 4.44 and 84.50 ± 5.15 *μ*m in the controls, respectively (*P* = 0.04 and *P* = 0.03, resp.). The other GCIPL thicknesses were not significantly different between the hydroxychloroquine patients and the control subjects (both *P* > 0.05).

**Figure 2:**
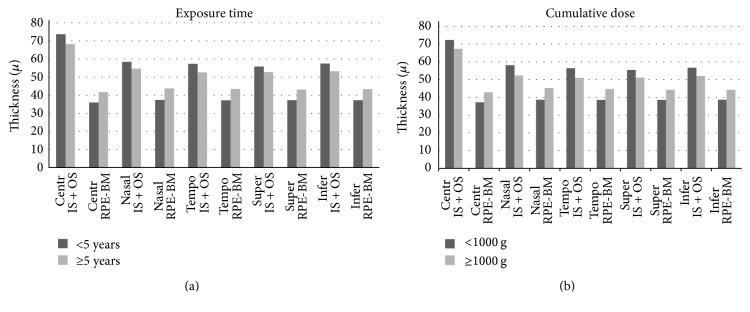
Bar graphs represent the photoreceptor total segment (IS + OS) thicknesses and retinal pigment epithelium- (RPE-) Bruch's membrane complex thicknesses; each one is noted according to the levels of the hydroxychloroquine exposure time values. These values are found to be statistically significant.

In the subgroup receiving hydroxychloroquine treatment ≥5 years, the IS + OS and OS thicknesses (except temporal) in the five measurement points were found to be lower and significantly different (*P* < 0.003) than in the subgroup treated with hydroxychloroquine <5 years. However, the IS thicknesses (except temporal) were not significantly different (*P* > 0.003). RPE-Bruch's membrane thicknesses for the group treated with hydroxychloroquine <5 years were also significantly higher than in the group receiving hydroxychloroquine ≥5 years (Table 4, Figure 2).

The GCIPL thickness values of the hydroxychloroquine patients are compared in Table 5. According to the results, the GCIPL thicknesses were not significantly different (*P* > 0.05). However, the subgroup treated with hydroxychloroquine ≥5 years had thinner mean GCIPL thicknesses than the subgroup receiving hydroxychloroquine treatment <5 years in all subfields.

The cumulative dose values were negatively correlated with the IS + OS thicknesses and positively correlated with RPE-Bruch's membrane complex thicknesses. These values are found to be statistically significant (*P* < 0.05) (Table 6).

## 4. Discussion

Hydroxychloroquine retinopathy has been well described and is one of the serious side effects associated with the use of hydroxychloroquine. Ocular screening tests have an important role in the early diagnosis and the prevention of drug toxicity [9]. The American Academy of Ophthalmology recommendations for screening that were published in 2016 recommended the use of both automated visual fields and SD-OCT for routine primary screening [2]. If signs of damage are uncertain and drug exposure continues, retinal degeneration and ultimately functional blindness may occur. In these conditions, early recognition and cessation of exposure clearly reduce the risk of later functional visual loss [10]. Cessation of the use of hydroxychloroquine at an early stage of damage might prevent functional loss; however, after maculopathy has developed, cessation of the drug does not show clinical recovery [6]. Because discontinuation of therapy may reverse retinal toxicity, early detection of toxicity changes is vitally important [11].

The risk factors for hydroxychloroquine retinopathy include daily dose adjusted for the lesser of IBW and ABW, duration of use more than 5 years, more than 1000 g total hydroxychloroquine consumption, increased age, concomitant renal or liver disease, and preexisting maculopathy [12, 13]. Therefore, 6.5 mg/kg/d based on IBW has been transformed from the threshold for safe dosing to the threshold for toxic dosing. Although hydroxychloroquine dosing should be based on the lesser of IBW and ABW, there is no consensus about the definition of IBW [14]. In our study, hydroxychloroquine dosing had been prescribed by the rheumatologist based on actual body weight. The given usual 400 mg/d dose of hydroxychloroquine would exceed the toxic dose threshold based on adjusted daily dose. Our calculated dose of hydroxychloroquine based on adjusted daily dosing, 8.75 mg/kg/d, is now considered too high.

The practical purpose of screening is to determine the earliest signs at a stage where visual loss is minimal or even asymptomatic. The most commonly available screening tests are VF assessments and SD-OCT. Although VF testing is subjective and variable, SD-OCT is objective and specific in providing retinal images [9, 15]. More certain diagnostic methods or the combination of objective and subjective tests can allow early-stage detection of retinal toxicity. SD-OCT was emphasized as one of the recommended screening methods for the early detection of hydroxychloroquine toxicity, according to the revised American Academy of Ophthalmology guidelines [13]. In a study, paracentral ring scotoma is documented in a few patients with early hydroxychloroquine retinal toxicity who had ring scotomas on VF testing but normal SD-OCT scans. The other patients in the same study had evident, parafoveal damage on SD-OCT images, accompanied by VF abnormalities [12]. In our study, quantitative analysis with segmentation presented significant thinning of the OS layers but VF testing did not show changes consistent with any retinopathy.

Hydroxychloroquine toxicity was reported in many previous studies using the SD-OCT. It has been indicated that OCT findings include loss of the external limiting membrane, disruption of the outer EZ, parafoveal thinning of the outer nuclear layer, and RPE damage [11, 16]. Our study is similar to previous reports and finds a significant decrease of the OS and IS + OS thicknesses in the patients using hydroxychloroquine. Moreover, the obtained data indicated a negative correlation with exposure time and cumulative dose. Likewise, in the subgroup receiving hydroxychloroquine treatment ≥5 years, the IS + OS and OS thicknesses (except temporal) in the five measurement points were found to be lower and significantly different than in the subgroup treated with hydroxychloroquine <5 years. Another observation in our study was that the minimum and temporal-inferior GCIPL thicknesses are significantly thinner in the group of patients using hydroxychloroquine compared to the control group. There was a slight decrease in the other GCIPL measurements, though this was not statistically significant. More specifically, Korah and Kuriakose [17] described these changes as anatomical evidence of loss of ganglion cell layers, causing marked thinning of the parafoveal region, especially in inferior and temporal quadrants. Previous histopathological studies reported that the initial dramatic changes of hydroxychloroquine toxicity were observed in the retinal ganglion cell layer [18, 19]. On the other hand, recent OCT studies have suggested that selective thinning of perifoveal inner plexiform and ganglion cell layers was found in patients treated with long-term hydroxychloroquine. These reported findings are in agreement with our observations [6, 20, 21].

We also observed that there was increased thickness of RPE-Bruch's membrane complex in hydroxychloroquine patients. Additionally, a positive correlation was found between exposure time and cumulative dose and an increase in thickness in RPE-Bruch's membrane. Similarly, RPE-Bruch's membrane thicknesses for the group treated with hydroxychloroquine <5 years were also significantly higher than in the group receiving hydroxychloroquine ≥5 years. Hydroxychloroquine binds to melanin in the RPE and causes degenerative changes. These changes in RPE metabolism result in a deterioration of the phagocytic function in the outer segments of the photoreceptor cells [22, 23]. Mahon et al. [24] reported an apparent accumulation of autophagic granules in cone photoreceptor cells exposed to chloroquine in an animal model. Pikkel et al. [25] indicated increased thicknesses of the outer band of the retina in the macular area in patients who were treated with hydroxychloroquine, compared to the control subjects. They supposed that the thickening of the outer band observed in patients resulted from thickening of Bruch's membrane. We think that an increase in RPE-Bruch's membrane complex thickness does not necessarily mean that the patient is experiencing toxicity. Therefore, we offer that a follow-up study examining patients who develop toxicity over time is needed. In the literature, the central thickness is affected only at the late stage to the fovea, while in patients with hydroxychloroquine toxicity, retinal thinning begins in a parafoveal ring and progresses over time to the outer parafoveal ring. Mititelu et al. [26] have noted that ELM integrity in or around the fovea on SD-OCT is associated with the preservation and possibility of EZ regeneration. In addition, they detected that patients with clinically visible disruption of the ELM during the initial examination had evidence of progressive outer retinal remodeling on SD-OCT after discontinuing hydroxychloroquine therapy. Specifically, the improvement in appearance of the outer retina and the partial regeneration of photoreceptors were limited to areas with ELM preservation at the time of diagnosis. Finally, they concluded that ELM preservation may carry a positive prognostic value for restorating the outer retinal layers in the setting of toxic effects.

Our study had several limitations. First, this study did not have a normal control group composed of patients with rheumatological disease not taking hydroxychloroquine. Our results may be reexamined in a future investigation involving more age-matched, untreated rheumatic of subjects as a control group. Second, we did not evaluate additional useful screening tests as the mfERG and fundus autofluorescence (FAF). In the previous studies, mfERG has been shown to be abnormal in retinal function even earlier than other modalities in patients with otherwise normal clinical examinations. It may be able to detect subtle changes in earlier stages of hydroxychloroquine toxicity [27, 28]. Currently, the American Academy of Ophthalmology recommendations for screening reported that the mfERG can provide objective corroboration for visual fields, and fundus autofluorescence (FAF) can show damage topographically [2]. However, mfERG testing is not available in most ophthalmology practices and requires specialized training to perform and analyze the test results. The present study would support the need for further analysis of the purported benefits of use as routine OCT and 10-2 VF testing [29]. Finally, the thicknesses were measured at 0.5 mm diameter, centered on the fovea. The segmentation was found to be rather poor because the OCT images were not clear to identify the origin of the outer retinal layers outside of the fovea. Therefore, we cannot measure at 1 or 1.5 mm from the fovea. Despite significant study results, there is the need for further studies to ascertain the relationship between changes in this photoreceptor segment and RPE-Bruch membrane thicknesses and the long-term risk of retinal toxicity with hydroxychloroquine treatment. We hope that future studies would improve our understanding of the value of this evaluation.

In conclusion, this study indicated that the foveal photoreceptor OS thinning, loss of GCIPL, and RPE-Bruch's membrane thickening were detected in patients with hydroxychloroquine therapy, using SD-OCT. We suppose that this quantitative approach using SD-OCT images may have important implications to use as an early indicator of hydroxychloroquine toxicity without any visible signs of hydroxychloroquine retinopathy.

## Figures and Tables

**Table 1:** Demographic characteristics and clinical data of users of hydroxychloroquine and controls.

	Hydroxychloroquine users	Controls	*P* value
	Mean ± SD	Mean ± SD	
Subjects (*n*)	51	30	—
Eye (*n*)	51	30	—
Sex (male/female)	1/50	1/29	—
Age, y	45.0 ± 13.16	42.1 ± 14.3	0.35
IOP, mmHg	15.95 ± 1.32	16.17 ± 1.47	0.18
Axial length, mm	23.03 ± 0.66	23.03 ± 0.67	0.95
CCT, *µ*m	540.42 ± 32.90	546.28 ± 3.78	0.25
BMI, kg/m^2^	25.17 ± 4.12	24.64 ± 4.15	0.58
Cumulative dose, g	648.41 ± 512.32	NA	—
Duration of use, mo	55.25 ± 41.27	NA	—
Adjusted daily dose, mg/kg/d	8.75 ± 0.11	NA	—
Diagnosis of patients, *n* (%)			
Rheumatoid arthritis	20 (39.2)	NA	—
Systemic lupus erythematosus	19 (37.3)	NA	—
Sjögren's syndrome	12 (23.5)	NA	—

IOP: intraocular pressure; CCT: central corneal thickness; BMI: body mass index; SD: standard deviation; *P* value Student's *t*-test, *P* < 0.05.

**Table 2:** Comparison of SD-OCT image segmentation values in hydroxychloroquine use and controls.

Thickness (*µ*)	Hydroxychloroquine use	Control	
	Mean ± SD	Mean ± SD	*P* value^*∗*^
Central			
IS	29.84 ± 6.50	29.43 ± 5.10	0.768
OS	41.25 ± 6.95	48.80 ± 4.05	0.001
IS + OS	71.10 ± 6.73	78.23 ± 3.76	0.001
RPE-BM	38.63 ± 4.39	30.07 ± 2.15	0.001
Nasal			
IS	28.71 ± 4.36	29.93 ± 2.84	0.172
OS	27.98 ± 4.41	32.07 ± 2.85	0.001
IS + OS	56.69 ± 5.49	62.00 ± 2.13	0.001
RPE-BM	40.37 ± 4.37	31.53 ± 2.30	0.001
Temporal			
IS	28.37 ± 3.60	29.90 ± 3.09	0.055
OS	26.69 ± 4.31	31.67 ± 2.67	0.001
IS + OS	55.06 ± 4.94	61.57 ± 2.30	0.001
RPE-BM	40.12 ± 4.33	31.30 ± 2.44	0.001
Superior			
IS	28.00 ± 4.43	29.33 ± 2.31	0.130
OS	26.37 ± 4.50	32.03 ± 2.72	0.001
IS + OS	54.37 ± 5.80	61.37 ± 2.40	0.001
RPE-BM	39.98 ± 4.27	31.03 ± 2.24	0.001
Inferior			
IS	28.53 ± 3.75	29.77 ± 2.25	0.105
OS	26.96 ± 4.46	31.70 ± 2.84	0.001
IS + OS	55.49 ± 5.07	61.47 ± 2.16	0.001
RPE-BM	40.12 ± 4.14	31.13 ± 2.16	0.001

IS: inner segment thickness; OS: outer segment thickness; IS + OS: total segment thickness; RPE-BM: retinal pigment epithelium-Bruch's membrane complex thickness; SD: standard deviation.

^*∗*^ *P* value adjusted for all the comparisons listed in the table.

**Table 3:** Comparison of macular GCIPL thickness in hydroxychloroquine use and controls.

Thickness (*µ*)	Hydroxychloroquine use	Control	
	Mean ± SD	Mean ± SD	*P* value
Average	82.71 ± 5.26	84.23 ± 5.46	0.22
Minimum	79.73 ± 5.07	81.97 ± 4.44	0.04
Temporal-superior	80.20 ± 5.53	81.67 ± 4.97	0.23
Superior	82.86 ± 5.84	84.40 ± 5.54	0.25
Nasal-superior	84.20 ± 6.45	85.23 ± 6.65	0.49
Nasal-inferior	84.24 ± 5.56	85.40 ± 5.99	0.38
Inferior	82.73 ± 5.59	84.77 ± 5.54	0.12
Temporal-inferior	81.78 ± 5.44	84.50 ± 5.15	0.03

SD: standard deviation.

**Table 4:** Comparison of SD-OCT image segmentation values in subgroups based on the duration of hydroxychloroquine use.

	Hydroxychloroquine use		
Thickness (*µ*)	<5 years	≥5 years	
	Mean ± SD	Mean ± SD	*P* value^*∗*^
Central			
IS	30.85 ± 5.76	28.71 ± 7.19	0.243
OS	42.81 ± 6.20	39.50 ± 7.45	0.089
IS + OS	73.67 ± 6.39	68.21 ± 5.99	0.001
RPE-BM	35.93 ± 2.81	41.67 ± 3.84	0.001
Nasal			
IS	29.56 ± 3.86	27.75 ± 4.78	0.141
OS	28.89 ± 4.27	26.96 ± 4.43	0.119
IS + OS	58.44 ± 4.86	54.71 ± 5.57	0.001
RPE-BM	37.41 ± 2.50	43.71 ± 3.53	0.001
Temporal			
IS	29.70 ± 3.07	26.88 ± 3.62	0.001
OS	27.59 ± 4.60	25.67 ± 3.80	0.112
IS + OS	57.30 ± 4.49	52.54 ± 4.22	0.001
RPE-BM	37.19 ± 2.63	43.42 ± 3.37	0.001
Superior			
IS	28.89 ± 4.66	27.00 ± 4.02	0.129
OS	26.96 ± 4.93	25.71 ± 3.97	0.325
IS + OS	55.85 ± 6.01	52.71 ± 5.17	0.052
RPE-BM	37.22 ± 3.07	43.08 ± 3.17	0.001
Inferior			
IS	29.00 ± 3.77	28.00 ± 3.74	0.347
OS	28.48 ± 4.49	25.25 ± 3.82	0.008
IS + OS	57.48 ± 5.15	53.25 ± 3.99	0.001
RPE-BM	37.26 ± 2.64	43.33 ± 3.00	0.001

IS: inner segment thickness; OS: outer segment thickness; IS + OS: total segment thickness; RPE-BM: retinal pigment epithelium-Bruch's membrane complex thickness; SD: standard deviation. Analysis was performed using Student's *t*-test.

^*∗*^ *P* value adjusted for all the comparisons listed in the table.

**Table 5:** Comparison of macular GCIPL thickness in subgroups based on the duration of hydroxychloroquine use.

Thickness (*µ*)	Hydroxychloroquine use		
	<5 years	≥5 years	
	Mean ± SD	Mean ± SD	*P* value
Average	83.74 ± 4.62	81.54 ± 5.78	0.14
Minimum	80.48 ± 4.91	78.88 ± 5.22	0.26
Temporal-superior	81.00 ± 4.90	79.29 ± 6.14	0.28
Superior	83.93 ± 5.05	81.67 ± 6.53	0.17
Nasal-superior	85.04 ± 5.56	83.25 ± 7.33	0.33
Nasal-inferior	85.04 ± 5.26	83.33 ± 5.85	0.28
Inferior	84.07 ± 5.06	81.21 ± 5.87	0.07
Temporal-inferior	83.07 ± 5.29	80.33 ± 5.35	0.07

*P* value Student's *t*-test.

**Table 6:** Results of Pearson's correlation analysis in users of hydroxychloroquine according to cumulative dose.

Thickness (*µ*)	Cumulative dose	
	*r*	*P* value^*∗*^
Central		
IS + OS	−0.41	0.002
RPE BM	0.63	0.001
Nasal		
IS + OS	−0.41	0.003
RPE BM	0.71	0.001
Temporal		
IS + OS	−0.51	0.001
RPE BM	0.70	0.001
Superior		
IS + OS	−0.29	0.041
RPE BM	0.63	0.001
Inferior		
IS + OS	−0.38	0.006
RPE BM	0.66	0.001

IS + OS thickness, photoreceptor inner (IS) and outer (OS) segment, and sum of them (IS + OS); RPE-BM complex, retinal pigment epithelium-Bruch's membrane complex.

Pearson's correlation coefficient (*r*).

^*∗*^ *P* value adjusted for all the comparisons listed in the table.
